# The development and initial validation of IgG4-related disease damage index: a consensus report from Chinese IgG4-RD Consortium

**DOI:** 10.1136/rmdopen-2023-003938

**Published:** 2024-03-08

**Authors:** Linyi Peng, Jingna Li, Jiaxin Zhou, Yunyun Fei, lingli Dong, Yan-Ying Liu, Dingding Zhang, Yanhong Wang, Shuhong Chi, Fang Wang, Yunxia Hou, Xiaoping Hong, Hongsheng Sun, Yujin Ye, Nan Che, Rong Zhang, Changyan Liu, Zongfei Ji, Wenjia Sun, Cheng Zhao, Ning Ma, Yamin Lai, Mengtao Li, Yan Zhao, Xiaofeng Zeng, Liwei Lu, Wen Zhang

**Affiliations:** 1 Department of Rheumatology and Clinical Immunology, Peking Union Medical College Hospital, Chinese Academy of Medical Sciences & Peking Union Medical College, National Clinical Research Center for Dermatologic and Immunologic Diseases (NCRC-DID), State Key Laboratory of Complex Severe and Rare Diseases, The Ministry of Education Key Laboratory, Beijing, China; 2 Department of Rheumatology and Immunology, Tongji Hospital, Tongji Medical College, Huazhong University of Science and Technology, Wuhan, Hubei, China; 3 Department of Rheumatology and Immunology, Beijing Friendship Hospital, Capital Medical University, Beijing, Beijing, China; 4 Medical Research Center, State Key laboratory of Complex Severe and Rare Diseases, Peking Union Medical College Hospital, Chinese Academy of Medical Sciences and Peking Union Medical College, Beijing, China; 5 Department of Epidemiology and Bio-statistics, Institute of Basic Medical Sciences, Chinese Academy of Medical Sciences & School of Basic Medicine, Peking Union Medical College, Beijing, China; 6 Department of Rheumatology, General Hospital of Ningxia Medical University, Yinchuan, China; 7 Department of Rheumatology, Beijing Hospital, Beijing, China; 8 Department of Rheumatology, The Affiliated Hospital of Inner Mongolia Medical University, Hohhot, China; 9 Department of Rheumatology and Immunology, The Second Clinical Medical College of Jinan University, The First Affiliated Hospital of Southern University of Science and Technology, Shenzhen People’s Hospital, Shenzhen, Guangdong, China; 10 Department of Rheumatology and Immunology, Shandong Provincial Hospital Affiliated to Shandong First Medical University, Jinan, China; 11 Department of Rheumatology, The First Affiliated Hospital of Sun Yat-Sen University, Guangzhou, China; 12 Department of Rheumatology, The First Affiliated Hospital of Nanjing Medical University, Nanjing, China; 13 Department of Rheumatology and Immunology, The First Affiliated Hospital of China Medical University, Shenyang, China; 14 Department of Rheumatology, the Second Affiliated Hospital of Dalian Medical University, Dalian, China; 15 Department of Rheumatology, Zhongshan Hospital, Fudan University, Shanghai, China; 16 Department of Rheumatology, The Second Affiliated Hospital Zhejiang University School of Medicine, Hangzhou, China; 17 Nanjing University Medical School Affiliated Nanjing Drum Tower Hospital, Nanjing, Jiangsu, China; 18 Department of Rheumatology, The First Hospital of Jilin University, Changchun, China; 19 Department of Gastroenterology, Peking Union Medical College Hospital, Chinese Academy of Medical Sciences & Peking Union Medical College, Beijing, China; 20 Department of Pathology and Shenzhen Institute of Research and Innovation, The University of Hong Kong, Hong Kong, China

**Keywords:** Autoimmune Diseases, Outcome Assessment, Health Care, Severity of Illness Index

## Abstract

**Objective:**

To develop and conduct an initial validation of the Damage Index for IgG4-related disease (IgG4-RD DI).

**Methods:**

A draft of index items for assessing organ damages in patients with IgG4-RD was generated by experts from the Chinese IgG4-RD Consortium (CIC). The preliminary DI was refined using the Delphi method, and a final version was generated by consensus. 40 IgG4-RD cases representing four types of clinical scenarios were then selected, each with two time points of assessment for at least 3 years of follow-up. 48 rheumatologists from 35 hospitals nationwide were invited to evaluate organ damage using the CIC IgG4-RD DI. The intraclass correlation coefficient (ICC) and the Kendall-W coefficient of concordance (KW) were used to assess the inter-rater reliability. The criterion validity of IgG4-RD DI was tested by calculating the sensitivity and specificity of raters.

**Results:**

IgG4-RD DI is a cumulative index consisting of 14 domains of organ systems, including a total of 39 items. The IgG4-RD DI was capable of distinguishing stable and increased damage across the active disease subgroup and stable disease subgroup. In terms of scores at baseline and later observations by all raters, overall consistency in scores at baseline and later observations by all raters was satisfactory. ICC at the two time points was 0.69 and 0.70, and the KW was 0.74 and 0.73, respectively. In subgroup analysis, ICC and KW in all subgroups were over 0.55 and 0.61, respectively. The analysis of criterion validity showed a good performance with a sensitivity of 0.86 (95% CI 0.82 to 0.88), a specificity of 0.79 (95% CI 0.76 to 0.82) and an area under the curve of 0.88 (95% CI 0.85 to 0.91).

**Conclusion:**

The IgG4-RD DI is a useful approach to analyse disease outcomes, and it has good operability and credibility. It is anticipated that the DI will become a useful tool for therapeutic trials and studies of prognosis in patients with IgG4-RD.

**WHAT IS ALREADY KNOWN ON THIS TOPIC**

- The degree of organ damage is a crucial factor affecting the life quality and long-term prognosis of patients with IgG4-related disease (IgG4-RD).

**WHAT THIS STUDY ADDS**

- IgG4-RD Damage Index was developed to evaluate persistent organ damage by a consensus report from Chinese IgG4-RD Consortium (CIC).
- The CIC IgG4-RD DI was performed by the standard of criterion validity and inter-rater reliability.

**HOW THIS STUDY MIGHT AFFECT RESEARCH, PRACTICE OR POLICY**

- It is anticipated that the CIC IgG4-RD DI will become a useful tool for therapeutic trials and studies of prognosis in patients with IgG4-RD.

## Introduction

IgG4-related disease (IgG4-RD) is a newly recognised chronic systemic fibroinflammatory disease with multiple organ involvement and can lead to irreversible organ damage, dysfunction or even death during the progression of the disease.^1 2^ In recent years, although significant progress has been achieved in the pathogenesis, clinical characteristics, diagnosis and treatment of IgG4-RD, there are still many unmet needs since the nomination of this disease.

Similar to other systemic autoimmune diseases, patients with IgG4-RD may develop irreversible organ damage with the fluctuation of disease activation and remission.^3 4^ Uncontrolled type I autoimmune pancreatitis (AIP-1) results in pancreatic dysfunction and consequent diabetes mellitus and malabsorption.^5 6^ IgG4-related cholangitis is often concurrent with AIP-1 and may cause cirrhosis and end-stage liver disease.^7^ Cardiovascular involvement in IgG4-RD includes periaortitis, periarteritis, phlebitis, pulmonary vascular disease, valvopathy, pericardial disease, myocardial disease, cerebrovascular disease and vasculitis.^8^ Among them, periaortitis is a major cause of inflammatory aneurysms and always has a poor prognosis.^9^ Urinogenital IgG4-RD typically manifests as interstitial nephritis, renal pelvis and ureter involvement, causing renal dysfunction, and complete recovery may be difficult.^10 11^ Although most patients with IgG4-RD respond well to glucocorticoid-based treatment, this disease inclines to relapse when glucocorticoid is tapered to a low dosage or cessation.^12–14^ In addition to the persistence or recurrence of the disease, long-term use of glucocorticoids or immunosuppressants can also lead to drug-related side effects as well as risks of organ damage. Moreover, patients with organ-occupying lesions who were initially suspected of tumour with surgical resection could also lead to irreversible organ damage and dysfunction. Thus, the degree of organ damage is a crucial factor affecting the life quality and long-term prognosis of patients with IgG4-RD.

Disease damage is an important surrogate for long-term outcomes in chronic autoimmune diseases such as systemic lupus erythematosus, Sjogren’s syndrome and systemic vasculitis. In the setting of chronic conditions, it becomes increasingly important to monitor the burden of disease in terms of both active inflammation and chronic damage (scarring) from primary disease and treatment, as well as disease-associated comorbidities. Clinical studies should be designed to decrease the amount of damage accumulating due to therapeutic intervention rather than simply controlling disease activity.^15^ Currently, the IgG4-RD Responder Index (RI) is a widely used tool for assessing disease activity and the efficacy of treatment in IgG4-RD research. Although IgG4-RD RI also includes assessments of disease damage in each domain, it only records the number of impaired organs without assessing the severity of organ damage. Moreover, damages due to medications or other treatments of IgG4-RD are not included in IgG4-RD RI.^16^ Therefore, there is an urgent need to develop a new Damage Index (DI) for evaluating organ damages in patients with IgG4-RD.

In this study, we aimed to design and validate an IgG4-RD DI to evaluate irreversible organ damage and dysfunction, which include disease-related and treatment-related damage. In the process of designing the scoring system, we referenced the DIs of systemic lupus erythematosus,^17 18^ Sjogren’s syndrome^19^ and systemic vasculitis,^20^ as well as IgG4-RD RI.^14^ We hope that this instrument can be used for assessing the prognosis and long-term disease burden of IgG4-RD.

## Methods

### Overview of the instrument development approach

The Chinese IgG4-RD Consortium (CIC) IgG4-RD DI was designed by a steering committee to assess organ damage from visit to visit using clinician-generated assessments of both disease symptoms and objective measures. The prototype of CIC IgG4-RD DI employed a scoring system of 14 domains, consisting of 13 organ systems and other damages that were not listed in the previous 13 organ systems, including disease-related damages, treatment-related adverse effects and malignancies. We defined DI score as referring to irreversible organ damage that has lasted at least 6 months since IgG4-RD was diagnosed. IgG4-RD DI score can only remain stable or deteriorate. As a newly recognised disease, large long-term follow-up cohort studies were still limited in IgG4-RD. It is difficult to build a prediction model to formulate the weight coefficients of different organs for prognosis, such as the correlation between organ damage and the endpoint of mortality. In CIC IgG4-RD DI, item weighting is established mainly according to expert consensus. The scores of some organs are superimposed to increase the weight coefficient of important organs. We attempted to set scores based on both functional and radiological assessments. For example, for vital organs such as the liver and kidneys, the maximum damage scores are 4 and 5 points, respectively.

The CIC IgG4-RD DI was revised by CIC members who gathered to perform a modified Delphi process in June 2021. This group was comprised of 43 physicians and 2 statisticians from 29 medical centres in China, with subspecialty expertise in rheumatology, gastroenterology, ophthalmology, nephrology, stomatology, pathology and radiology. It was agreed within the group to base the process on a questionnaire through social media network, online video conferences and open as well as closed ballots within the group. Each item in the questionnaire was voted on by experts to indicate the extent of their agreement. If the rating were lower than 80% agreement, it would be discussed and revised at the open conference accordingly.

### Testing of CIC IgG4-RD DI

#### Construction of clinical scenario cases of typical IgG4-RD

In order to validate the criterion and discrimination of CIC IgG4-RD DI, 17 centres participated in constructing clinical scenarios of typical IgG4-RD cases according to their real patients who had been diagnosed with IgG4-RD according to the 2019 American College of Rheumatology/EULAR Classification Criteria for IgG4-RD^2^ and/or the 2020 Revised Comprehensive Diagnostic Criteria for IgG4-RD^21^ and regularly followed up for at least 3 years. All these case scenarios were submitted to Peking Union Medical College Hospital, and 40 cases that represented 4 kinds of clinical scenarios were then selected into 4 groups and rewritten in a uniform format (online supplemental table 1): the first group (activve-increased), patients with active disease and increased damage; the second group (active-stable), patients with active disease and stable damage; the third group (stable-increased), patients with inactive diseases and increased damage; and the fourth group (stable-stable), patients with inactive disease and stable damage.

10.1136/rmdopen-2023-003938.supp2 Supplementary data

Four experts selected by the steering committee were responsible for evaluating the standard scores of disease activity and organ damage according to patients’ clinical symptoms, laboratory parameters, imaging results, complications and brief treatment processes. The time at which damage was assessed was marked on each case history, as time one and time two.

### Training and collection of scoring

48 rheumatologists from 35 hospitals nationwide were invited for a training course to evaluate organ damage by using the CIC IgG4-RD DI. Subsequently, they were asked to evaluate and score the DI of all 40 case scenarios, with each case at 2 different time points. The electronic forms were returned for further analysis.

### Statistical analysis

In the descriptive analysis, we used mean and SD or median and IQR for continuous variables according to data distribution. To assess the reliability of CIC IgG4-RD DI, both the intraclass correlation coefficient (ICC) and Kendall-W coefficient of concordance (KW) were calculated based on the scores of the 48 raters for the 40 cases. Furthermore, we calculated the difference between the first and second assessments of the 48 raters for the 40 cases. Fleiss’ Kappa was calculated for the 48 raters by classifying the differences into two groups (whether damage increased or stable). The bootstrap method was used to estimate the 95% CIs for ICC, KW and Fleiss’ Kappa with 2000 times of resampling. The R software (V.4.2.0) was employed to conduct the analysis with the packages irr, DescTools and boot.

To assess the criterion validity of CIC IgG4-RD DI, we used the expert score as the gold standard and divided the 40 cases into 2 groups (increased and stable damage). First, we compared the differences of the first and the second assessment for the 48 raters between the 2 groups using the Mann-Whitney U test. Second, we calculated the sensitivity and specificity of each rater based on the gold standard. Then, we calculated the summary operating sensitivity and specificity and plotted the summary receiver operating characteristic curve (SROC) with a bivariate method using the midas package in Stata 12.0. All the analysis treated two-sided p<0.05 as a statistically significant level.

## Results

### CIC IgG4-RD DI

The first edition of CIC IgG4-RD DI was initially discussed and revised by the members of the CIC; the consistency among experts on each domain is shown in table 1. 12 out of 14 domains of items proposed reached a consistency of over 80% and were approved. Among all domains, lower consistency came out to be damage to the lungs (77.08%) and others (75%). Preliminary items of damage to the lungs included radiological indications including pulmonary fibrosis and masses, pulmonary hypertension and lung dysfunction defined by forced vital capacity or forced expiratory volume in one second or single breath diffusing capacity for carbon monoxide <60%. Voters disagreed on lung function thresholds. In addition, there was controversy over the detailed definition of radiological findings of lung damage. After thorough discussions, the lung function cut-off values were adjusted. In terms of the others, experts diverged on whether chronic infections had causal links to IgG4-RD or medication and recommended the deletion of this item. Members later reviewed and accepted a final edition of the CIC IgG4-RD DI (table 2).

**Table 1:** Voting of the first version of the IgG4-RD DI by CIC

Domain	Agreement
1. Nervous system	95.85%
2. Pituitary	91.67%
3. Orbits	89.58%
4. Lacrimal/salivary gland	89.58%
5. Mastoid/auris media	95.83%
6. Nose/nasal sinus	87.50%
7. Thyroid gland	91.67%
8. Lung	77.08%
9. Cardiovascular system	89.58%
10. Retroperitoneum/mediastinum	95.83%
11. Pancreas	95.83%
12. Liver/biliary tree	83.33%
13. Kidney	87.50%
14. Others	75.00%

CIC, Chinese IgG4-RD Consortium; IgG4-RD DI, IgG4-related-disease Damage Index.

**Table 2:** Damage Index for IgG4-RD (IgG4-RD DI) by CIC

Item	Score
Nervous system	
Persistent/residual meninges thickening (imaging)	1
Cranial or peripheral nerve damage (not including optic nerve)	1
Pituitary gland	
Anterior pituitary dysfunction	1
Central diabetes insipidus	1
Orbits	
Visual impairment (if blindness, rate 2)	1 (2)
Diplopia or exophthalmos	1
Lacrimal/salivary gland^25^	
Dry mouth with objective evidence of xerostomia	1
Dry eyes with objective evidence of xerophthalmia	1
Mastoid/auris media	
Persistent mastoiditis (imaging)	1
Hearing loss	1
Nose/nasal sinus	
Persistent nasal sinus/turbinal lesions (imaging) with relevant clinical symptoms	1
Thyroid gland	
Hypothyroidism (exclusion of other causes)	1
Persistent enlargement of thyroid gland compressing bronchia	1
Lung	
Persistent lung fibrosis/pleural thickening/masses/bronchial lesion (imaging)^26^	1
Pulmonary hypertension	1
Impaired lung function (FVC <80% or FEV_1_ <80% or DLCO <70% predicted values)	1
Cardiovascular system	
Luminal stenosis, or formation of aneurysms (imaging), or in need of interventional procedures or surgery	1
Constrictive pericarditis	1
Retroperitoneum/mediastinum	
Persistent retroperitoneal or mediastinal masses	1
Hydronephrosis/long-term D-J tube placement (more than 6 months)^10^	1
Pancreas	
Pancreatic atrophy, persistent enlargement or pseudocyst (imaging)	1
Long-term pancreatic enzyme replacement due to exocrine insufficiency	1
Liver/biliary tree	
Intrahepatic and/or extrahepatic biliary duct stricture (imaging)	1
Persistent elevation of liver enzyme/bile duct enzyme/bilirubin over two times the normal upper limit	1
Compensated cirrhosis (Child-Pugh grade A) or regional portal hypertension	1
Decompensated cirrhosis (Child-Pugh grade B/C)	2
Kidney	
Persistent renal parenchyma/pelvis masses (imaging)	1
24-hour urinary protein >0.5 g	1
eGFR 30–60 mL/(min×1.73 m^2^) (calculated by CKD-EPI)^27^	2
eGFR 15–30 mL/(min×1.73 m^2^) or need renal replacement therapy	3
Others	
Irreversible damage to other organs not listed above (accumulative if >one site)	1
New-onset malignancy (accumulative in case of more than one kind of malignancy)	1
Disease-related or treatment-related cerebrovascular/cardiovascular accident (if more than once, rate 2)	1 (2)
Persistent drug-related myelosuppression (more than 6 months)	1
Glucocorticoid-related ischaemic osteonecrosis	1
Glucocorticoid-related osteoporosis with fractures or vertebral collapse	1
Cataracts caused by glucocorticoids	1
Disease-related or treatment-related diabetes mellitus	1
Partial or total resection of organs due to IgG4-RD (accumulative if >one site), including but not limited to orbitotomy, pulmonary wedge resection, Whipple procedure, nephrectomy, pituitary surgery and thyroidectomy	1

CIC, Chinese IgG4-RD Consortium; CKD-EPI, the Chronic Kidney Disease Epidemiology Collaboration equation; DLCO, diffusing capacity of the lungs for carbon monoxide; eGFR, estimated glomerular filtration rate; FEV_1_, forced expiratory volume in one second; FVC, forced vital capacity; IgG4-RD DI, IgG4-related disease Damage Index.

The CIC IgG4-RD DI is a cumulative index consisting of 14 domains of organ systems involved in IgG4-RD, including a total of 39 items. The instruction manual of CIC IgG4-RD DI is shown in online supplemental appendixes 1 and 2. The DI emphasised persistent organ damage based on objective findings, such as radiological proof of damage to the pancreas, bile ducts, lungs, orbits, kidneys and pituitary glands. Persistent organ functional damage affecting patients’ quality of life was also considered. In addition, the DI also included damages caused by treatment, such as diabetes mellitus, cataracts, osteoporosis, femoral head necrosis and drug-related myelosuppression. New onset malignancies were considered as well.

10.1136/rmdopen-2023-003938.supp1 Supplementary data

### Confirmation of test cases

**Figure 1:**
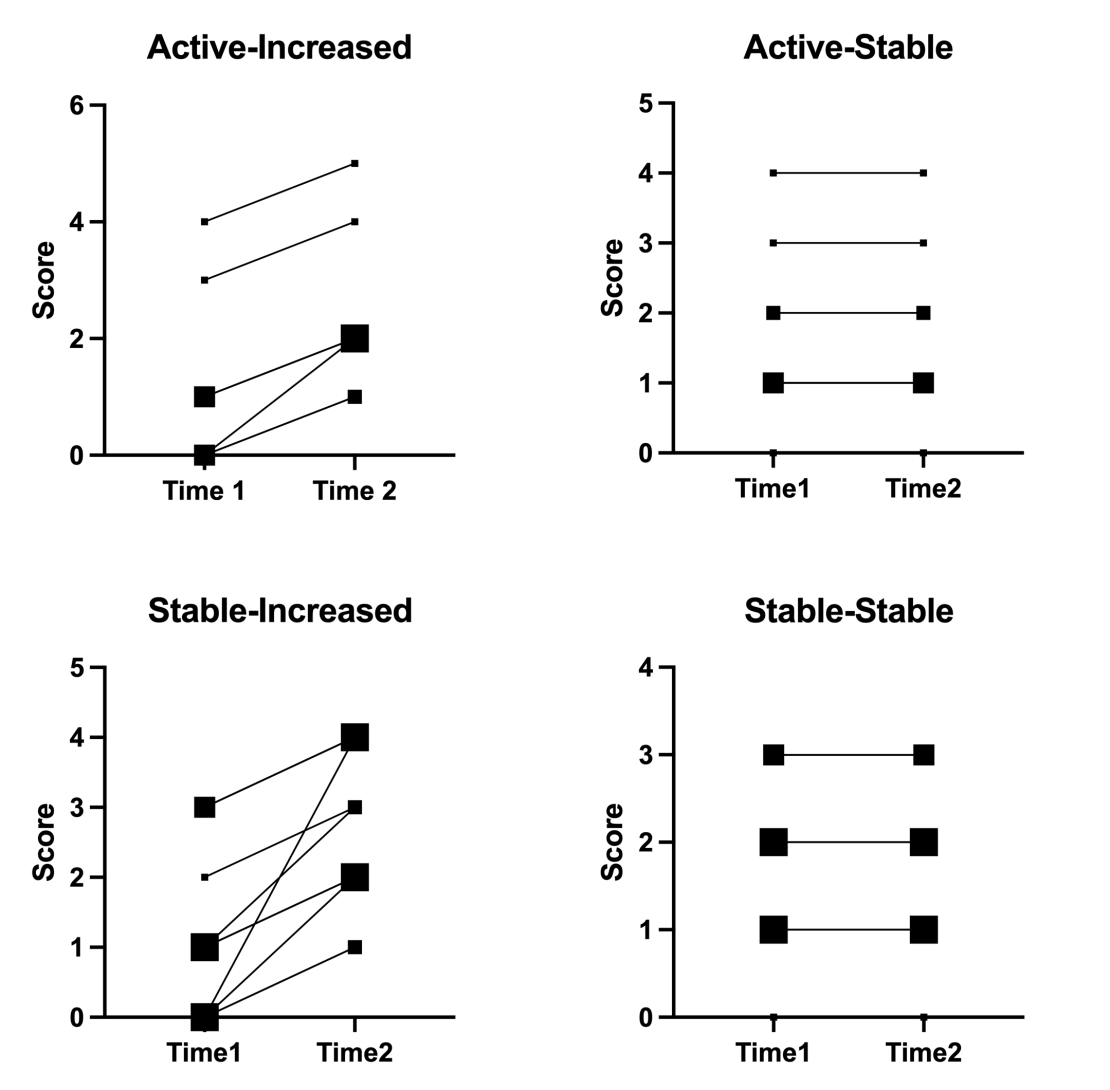
Overview of IgG4-RD DI scores of standard case scenario. Scores by experts of the steering committee were deemed standard scores. The size of the dots represents the number of patients with the same score.

40 test cases representing 4 clinical scenarios (active-increased, active-stable, stable-increased and stable-stable) were provided by 17 different centres. The DI scores which were confirmed by the steering committee members were recognised as the standard CIC IgG4-RD DI score. As shown in figure 1, the DI was capable of distinguishing damage stable and damage increased affected by both IgG4-RD itself and the adverse effect due to treatment across the active disease subgroup and stable disease subgroup. Scores increased in active-increased and stable-increased groups, while remaining unchanged in active-stable and stable-stable groups.

### Inter-rater reliability

The CIC IgG4-RD DI and 40 case scenarios were handed out to 48 trained raters who were rheumatology clinicians from 35 tertiary hospitals across the country. Scores of baselines and later observations were first used to calculate ICC and KW of both time points of scoring overall and by subgroups to assess consistency among raters. Overall consistency was satisfying. ICC at the two time points was 0.69 and 0.70, and the KW was 0.74 and 0.73, respectively. In subgroup analysis, ICC and KW in all subgroups were over 0.55 and 0.61, respectively. ICC and KW between raters were the best in the active-increased group at baseline, 0.84 and 0.86 (table 3). For further analysis, a subtraction of scores at time points 2 and 1 was made to evaluate the consistency of score changes between raters. We classified a score change ≥1 as ‘increased’ and 0 as ‘stable’. Fleiss’ Kappa was 0.50 (95% CI 0.393 to 0.506, p<0.001) demonstrating that the CIC IgG4-RD DI achieved satisfying inter-rater reliability.

**Table 3:** Assessment of the reliability of CIC IgG4-RD DI by interclass correlation coefficient (ICC) and Kendall-W coefficient of concordance (KW)

Group	Tie	ICC				KW			
		ICC	95% CI lower limit	95% CI upper limit	P value	KW	95% CI lower limit	95% CI upper limit	P value
Overall	1	0.69	0.59	0.79	<0.001	0.74	0.68	0.75	<0.0001
Active-increased	1	0.84	0.68	0.96	<0.0001	0.86	0.85	0.94	<0.0001
Active-stable	1	0.69	0.48	0.90	<0.0001	0.72	0.61	0.87	<0.0001
Stable-increased	1	0.68	0.51	0.86	<0.0001	0.74	0.67	0.82	<0.0001
Stable-stable	1	0.55	0.37	0.79	<0.0001	0.61	0.60	0.77	<0.0001
Overall	2	0.70	0.61	0.80	<0.001	0.73	0.67	0.75	<0.0001
Active-increased	2	0.67	0.46	0.89	<0.0001	0.67	0.53	0.74	<0.0001
Active-stable	2	0.69	0.48	0.92	<0.0001	0.72	0.65	0.83	<0.0001
Stable-increased	2	0.74	0.58	0.89	<0.0001	0.78	0.78	0.86	<0.0001
Stable-stable	2	0.64	0.44	0.85	<0.0001	0.67	0.73	0.89	<0.0001

### Criterion validity

**Figure 2:**
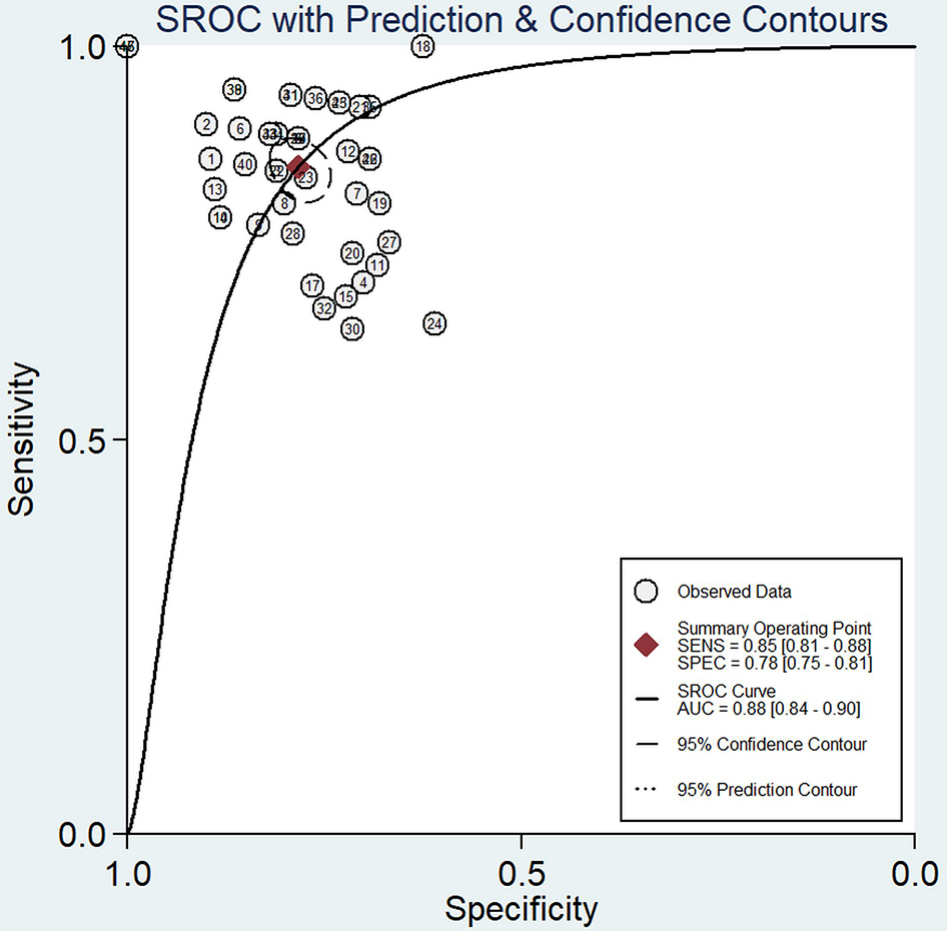
The summary receiver operating characteristic curve (SROC) of all raters. AUC, area under the curve. SENS, sensitivity. SPEC, specificity.

We established the gold standard for our study by using the scores of rater #1, who represented the consensus of core expert group of main experts involved in the development of CIC IgG4-RD DI and coauthored the simulation cases. The gold standard divided the clinical scenario cases into two groups: a damage increased group and a stable group. The scores of all raters could reflect the difference in the injury scores between the two groups, p<0.001 (online supplemental table 2). The sensitivity and specificity of each rater were calculated (online supplemental table 3). According to the SROC (figure 2), the sensitivity and specificity of the summary operating point were 0.86 (95% CI 0.82 to 0.88) and 0.79 (95% CI 0.76 to 0.82), respectively, and area under the curve was 0.88 (95% CI 0.85 to 0.91).

## Discussion

As IgG4-RD is a chronic inflammatory fibrotic disease, it is clear and important that accumulated damage should be evaluated besides disease activity for assessment of treatment strategy and long-term prognosis. We here described the philosophy and goals of the IgG4-RD DI. Each item of the DI was voted on and refined by members of experts of the CIC. The instrument was applied to scenarios of IgG4-RD patients for inter-rater reliability tests among trained raters from different centres nationwide. The initial validation suggests that the CIC IgG4-RD DI is a sensitive, reproducible, comprehensive and credible clinical instrument for recording the accumulation of damage in patients with IgG4-RD.

The widely used disease activity index, IgG4-RD RI, was first established in 2012^16^ and then revised twice in 2015 and 2018, respectively.^14 22^ It provided a useful evaluation tool for disease activity and treatment response of IgG4-RD, which marked an important step towards the availability of outcome measures in this disease. Besides disease activity, IgG4-RD RI also included organ damage evaluation which counts the number of irreversibly damaged organs. Compared with IgG4-RD RI, the CIC IgG4-RD DI is specified on organ damage with more items, and damages due to medications or other treatments of IgG4-RD are also included, as well as new-onset malignancies. It is important to compare the efficiency and credibility of the damaged part in IgG4-RD RI with the CIC IgG4-RD DI in future studies.

In CIC IgG4-RD DI, we attempted to distinguish certain coefficients based on experts’ clinical experience. Ideally, a DI should have a weighted score according to the organ systems involved and the severity of organ involvement. We also referred to the Systemic Lupus International Collaborating Clinics Systemic Lupus Erythematosus Damage Index (SLICC SLE-DI) and the Vasculitis Damage Index (VDI). It has been proved that there was no important improvement to SLICC SLE-DI after item weighting.^23^ The VDI was derived from SLICC SLE-DI, boasting more items than SLICC SLE-DI (67 vs 40). The VDI also did not weigh items while achieving more sensitivity.^20^ More cohort studies on the outcome of IgG4-RD are needed to help improve the conception of IgG4-RD DI in the future.

We also have to solve some disputes and problems among assessors in the formulation of scoring CIC IgG4-RD DI. For example, in assessing damage to the nose and nasal sinus, we included clinical symptoms such as difficulties in breathing, nasal discharge and pain, which may affect patients’ prognosis and quality of life. IgG4-RD-related retroperitoneal fibrosis (RPF) and mediastinal masses often could not disappear completely after treatment by imaging examinations, even with a good response to treatment. Therefore, we reached a consensus after discussion that if RPF and/or mediastinal masses significantly reduced after therapy and without compression of the surrounding organs, they should not be scored in DI. According to our previous study, patients with RPF commonly remove double-J catheter within 6 months of treatment; hence, we determined damage to the retroperitoneum as double-J catheter drainage for over 6 months.^10^ The score of the lungs is also controversial. The imaging manifestations of linear or reticular appearances, nodules, thickened bronchovascular bundles and pleural thickening, which do not affect pulmonary function, should not be included in the DI. Considering that the lung is an important organ and referring to Sjogren’s Syndrome DI,^19^ a total area of persistent lung fibrosis >10% of the lung field will be scored. Although the lymph node is a common organ involved in IgG4-RD, in consideration of the little impact on patients’ prognosis and quality of life, it is not included and scored in IgG4-RD DI. There is another concern; we define damage as irreversible changes over 6 months, but occasionally some lesions may still reduce after 6 months of treatment, which can cause a change in the score. In this condition, the previous damage score should be corrected as we should follow the principle that cumulative damage will not decrease.

Although there is controversy over the correlation between IgG4-RD and tumour, yet an accompaniment of the two entities, especially in the later course of IgG4-RD, is relatively common.^24^ Malignancies as disease damage were also previously listed in SLICC SLE-DI, Sjogren’s Syndrome DI and VDI.^17 19 20^ Given that malignancies could significantly impact patients’ prognosis, we decided to include newly onset malignancies in the scoring system.

The reliability or reproducibility was examined using the ICC and KW; the value of the whole 40 cases and each subgroup is all above 0.6, indicating that the CIC IgG4-RD DI score has a good reliability. The test of criterion validity showed that the damage scoring system had good sensitivity (0.85) and specificity (0.79), which indicated the DI tool could help clinicians to effectively distinguish whether the damage is increased. To achieve better consistency, it is necessary to give assessors a training course. The training course on clinical vignettes illustrated some shortcomings in the early version of the CIC IgG4-RD DI and simulation cases that led to appropriate revisions. Based on the disputed scoring points, we give definitions and instructions in online supplemental appendix to ensure the quality of scoring. New assessors are advised to carefully study the guidelines for the application of the CIC IgG4-RD DI. The DI includes 14 domains; both a thorough understanding of the clinical breadth of IgG4-RD itself and a high degree of familiarity with the index are required for its effective employment.^15^

There are some limitations in this study. First, unlike SLE and systemic vasculitis, distinguishment between the active state and cumulative damage of IgG4-RD is more difficult. Though we defined damage score as irreversible tissue damage lasting at least 6 months according to DIs of other diseases, it remains to be verified whether it is necessary to set a longer time for observation in IgG4-RD. Second, the CIC IgG4-RD DI was based on Chinese patients, and this DI should be validated in patients from countries other than China.

In conclusion, CIC IgG4-RD DI based on the consensus of experts from CIC is a useful approach to analysing disease outcomes, which has good operability and credibility. Our results have indicated that quantitative assessment of organ damage caused by disease and treatment will promote the effective evaluation of the prognosis of IgG4-RD.

## Data Availability

All data relevant to the study are included in the article or uploaded as supplementary information.
