# Supplementary material for: The development and initial validation of IgG4-related disease damage index: a consensus report from Chinese IgG4-RD Consortium
Source: RMD Open. 2024 Mar 8;10(1):e003938. doi: 10.1136/rmdopen-2023-003938 (PMC10928742; doi:10.1136/rmdopen-2023-003938)
Supplement: Supplementary data [file rmdopen-2023-003938supp002.pdf]

Supplemental Table 1

Case	Gender	Age	Disease duration before treatment	Time point of assessment after treatment (years)		Organ Damage		Damage Score in IgG4-RD DI		Damage score in IgG4-RD RI		Subgroup
				First assessment	Second assessment	First assessment	Second assessment	First assessment	Second assessment	First assessment	Second assessment	
**case1**	female	55	2yrs	0.5	3.5	None	None	0	0	0	0	Disease Active-Damage Stable
**case2**	female	60	1.5yrs	0.5	5	None	Salivary/lacrimal glands: Dry eyes with objective evidence of xerophthalmia	0	1	0	0	Disease Stable-Damage Increase
**case3**	male	57	9m	1	6	None	None	0	0	0	0	Disease Stable-Damage Stable
**case4**	female	80	1.5yrs	1	4	Kidney: eGFR 30-60ml/(min·1.73m²) (calculated by CKD-EPI)<br>Retroperitoneum: Hydronephrosis<br>Others: Glucocorticoids-related Osteoporosis with fractures or vertebral collapse	Kidney: eGFR 30-60ml/(min·1.73m²) (calculated by CKD-EPI)<br>Retroperitoneum: Hydronephrosis<br>Others: Glucocorticoids-related Osteoporosis with fractures or vertebral collapse	3	3	2	2	Disease Stable-Damage Stable
**case5**	female	54	4yrs	**1**	**6**	Nervous system: Persistent/residual meninges thickening (Imaging)<br>Mastoid/auris media: Persistent mastoiditis (Imaging), Hearing loss<br>Other: Irreversible damage to other organs not listed above (Abnormal signals in the right temporal and occipital lobes in head MRI)	Nervous system: Persistent/residual meninges thickening (Imaging)<br>Mastoid/auris media: Persistent mastoiditis (Imaging), Hearing loss<br>Other: Irreversible damage to other organs not listed above (Abnormal signals in the right temporal and occipital lobes in head MRI)	4	4	3	3	Disease Active-Damage Stable
**case6**	male	67	1.5yrs	1.25	5	Pancreas	Pancreas	1	1	1	1	Disease Active-Damage Stable
**case7**	male	26	3yrs	0.5	5	None	Pituitary gland: Central diabetes insipidus, anterior pituitary dysfunction	0	2	0	2	Disease Active-Damage Increase
**case8**	female	57	2m	1	4	Pancreas	Pancreas	1	1	1	1	Disease Active-Damage Stable
**case9**	male	77	1yr	**0.5**	**4**	Orbits: Diplopia or exophthalmos	Orbits: Diplopia or exophthalmos<br>Other: Disease-related or treatment-related cerebrovascular accident	1	2	1	1	Disease Stable-Damage Increase
**case10**	female	33	7yrs	**0.5**	**3.5**	Lacrimal/salivary gland: Dry eyes with objective evidence of xerophthalmia	Lacrimal/salivary gland: Dry eyes with objective evidence of xerophthalmia<br>Lung: Persistent bronchial lesion (Imaging)	1	2	1	2	Disease Stable-Damage Increase
**case11**	female	51	5yrs	0.5	3.5	Nasal sinus<br>Pituitary gland: Central diabetes insipidus<br>Kidney: Persistent renal parenchyma masses (Imaging)	Nasal sinus<br>Pituitary gland: Central diabetes insipidus<br>Kidney: Persistent renal parenchyma masses (Imaging)	3	3	3	3	Disease Active-Damage Stable
**case12**	male	54	3yrs	**1**	**5**	Lung: Persistent lung fibrosis (Imaging), Impaired lung function	Lung: Persistent lung fibrosis (Imaging), Impaired lung function	2	2	1	1	Disease Stable-Damage Stable
**case13**	male	62	6m	**0.5**	**3.5**	None	Other: Disease-related or treatment-related cerebrovascular accident	0	1	0	2	Disease Stable-Damage Increase
**case14**	female	33	7yrs	**1**	**4**	None	Lacrimal/salivary gland	0	1	0	1	Disease Active-Damage Increase
**case15**	male	69	4yrs	**3**	**6**	Lung: Persistent lung fibrosis/ pleural thickening /masses/bronchial lesion (Imaging)<br>Other: Glucocorticoids-related Ischemic osteonecrosis	Lung: Persistent lung fibrosis/ pleural thickening /masses/bronchial lesion (Imaging)<br>Other: Glucocorticoids-related Ischemic osteonecrosis, new-onset malignancy	2	3	1	1	Disease Stable-Damage Increase

**case16**	male	66	1yr	**1**	**4**	Lung: Persistent lung fibrosis (Imaging)	Lung: Persistent lung fibrosis (Imaging)<br>Lacrimal/salivary gland: Dry mouth with objective evidence of xerostomia, dry eyes with objective evidence of xerophthalmia	1	3	3	3	Disease Stable-Damage Increase
**case17**	female	72	11m	**1**	**3**	Lung: Persistent lung fibrosis (Imaging)<br>Kidney: eGFR 15-30ml/(min·1.73m^2)	Lung: Persistent lung fibrosis (Imaging)<br>Kidney: eGFR 15-30ml/(min·1.73m^2)	2	2	2	2	Disease Stable-Damage Stable
**case18**	male	53	3m	**0.5**	**5**	Pancreas: Pancreatic atrophy (Imaging), Long-term pancreatic enzyme replacement due to exocrine insufficiency<br>Other: Disease-related diabetes mellitus	Pancreas: Pancreatic atrophy (Imaging), Long-term pancreatic enzyme replacement due to exocrine insufficiency<br>Other: Disease-related diabetes mellitus	3	3	1	1	Disease Stable-Damage Stable
**case19 ***	male	47	1yr	**0.5**	**3.5**	Other: Disease-related or treatment-related diabetes mellitus	Pancreas: Pancreatic persistent enlargement (Imaging)<br>Other: Disease-related or treatment-related diabetes mellitus	1	2	3	3	Disease Active-Damage Increase
**case20**	female	54	8m	**0.5**	**3.5**	Nasal sinus	Nasal sinus	1	1	3	3	Disease Active-Damage Stable
**case21**	male	58	6m	**1**	**5**	Kidney: eGFR 30-60ml/(min·1.73m^2) (calculated by CKD-EPI)	Kidney: eGFR 30-60ml/(min·1.73m^2) (calculated by CKD-EPI)	1	1	1	1	Disease Stable-Damage Stable
**case22**	male	58	1m	**2**	**6**	Cardiovascular system: Formation of aneurysms (Imaging)<br>Other: Persistent drug-related myelosuppression	Cardiovascular system: Formation of aneurysms (Imaging)<br>Other: Persistent drug-related myelosuppression	2	2	2	2	Disease Stable-Damage Stable
**case23**	male	67	1yr	**5**	**8**	Lung: Persistent lung fibrosis, impaired lung function<br>Other: Disease-related or treatment-related diabetes mellitus	Lung: Persistent lung fibrosis, impaired lung function<br>Pancreas: Pancreatic persistent enlargement (Imaging)<br>Other: Disease-related or treatment-related diabetes mellitus	3	4	2	2	Disease Stable-Damage Increase
**case24**	male	55	3m	**0.5**	**5**	Orbits: Diplopia or exophthalmos, visual impairment	Orbits: Diplopia or exophthalmos, visual impairment	2	2	2	2	Disease Active-Damage Stable
**case25**	male	68	5yrs	**0.5**	**5**	None	Other: Cataracts caused by glucocorticoids, disease-related or treatment-related cardio-vascular accident	0	2	0	1	Disease Stable-Damage Increase
**case26**	female	53	2m	**0.5**	**3**	Lung: Persistent lung masses (Imaging)	Lung: Persistent lung masses (Imaging)	1	1	2	2	Disease Stable-Damage Stable
**case27**	male	74	1m	**0.5**	**4**	Retroperitoneum: Persistent retroperitoneal masses (Imaging), hydronephrosis	Retroperitoneum: Persistent retroperitoneal masses (Imaging), hydronephrosis	2	2	1	1	Disease Stable-Damage Stable
**case28**	male	67	4m	**0.5**	**5**	Lung: Persistent lung fibrosis/ pleural thickening /masses/bronchial lesion (Imaging)<br>Cardiovascular system: Luminal stenosis<br>Kidney: Persistent renal parenchyma masses (Imaging)	Lung: Persistent lung fibrosis/ pleural thickening /masses/bronchial lesion (Imaging)<br>Cardiovascular system: Luminal stenosis<br>Kidney: Persistent renal parenchyma masses (Imaging)<br>Other: Disease-related or treatment-related cerebrovascular accident	3	4	3	3	Disease Stable-Damage Increase
**case29**	male	78	6m	**2**	**5**	Lung: Persistent lung fibrosis (Imaging), impaired lung function<br>Retroperitoneum: Persistent retroperitoneal or mediastinal masses<br>Other: Irreversible damage to other organs not listed above	Lung: Persistent lung fibrosis (Imaging), impaired lung function<br>Retroperitoneum: Persistent retroperitoneal or mediastinal masses<br>Other: Irreversible damage to other organs not listed above, cataracts caused by glucocorticoids	4	5	3	3	Disease Active-Damage Increase
**case30**	male	65	17m	**0.5**	**5**	Pancreas: Pancreatic persistent enlargement (Imaging)<br>Liver/biliary tree: Intra- and/or extra-hepatic biliary duct stricture (Imaging)	Pancreas: Pancreatic persistent enlargement (Imaging)<br>Liver/biliary tree: Intra- and/or extra-hepatic biliary duct stricture (Imaging)	2	2	2	2	Disease Active-Damage Stable
**case31**	female	63	10yrs	**0.5**	**6**	Pancreas: Pancreatic atrophy (Imaging)<br>Liver/biliary tree: Intra- and/or extra-hepatic biliary duct stricture (Imaging), compensated cirrhosis (Child-Pugh grade A)	Pancreas: Pancreatic atrophy (Imaging), long-term pancreatic enzyme replacement due to exocrine insufficiency<br>Liver/biliary tree: Intra- and/or extra-hepatic biliary duct stricture (Imaging), compensated cirrhosis (Child-Pugh grade A)	3	4	2	2	Disease Active-Damage Increase
**case32**	female	67	3yrs	**0.5**	**3.5**	None	Other: Glucocorticoids-related Osteoporosis with fractures or vertebral collapse	0	1	0	1	Disease Active-Damage Increase

**case33**	male	55	1yr	**0.5**	**6**	None	Retroperitoneum: Persistent retroperitoneal masses, long-term D-J tube placement<br>Kidney: eGFR 30-60ml/(min·1.73m²) (calculated by CKD-EPI)<br>Other: Glucocorticoids-related Ischemic osteonecrosis	0	4	1	1	Disease Stable-Damage Increase
**case34**	male	26	3m	**0.5**	**5**	Thyroid gland	Thyroid gland	1	1	1	1	Disease Stable-Damage Stable
**case35**	male	77	1yr	**0.5**	**4**	Orbits	Orbits<br>Others: Disease-related or treatment-related cerebrovascular accident	1	2	1	1	Disease Active-Damage Increase
**case36**	male	29	1m	**0.5**	**7**	Kidney: eGFR 30-60ml/(min·1.73m²) (calculated by CKD-EPI)	Kidney: eGFR 30-60ml/(min·1.73m²) (calculated by CKD-EPI)<br>Other: Glucocorticoids-related Ischemic osteonecrosis	1	2	1	1	Disease Active-Damage Increase
**case37**	male	56	5yrs	**0.5**	**5**	Pancreas: Pancreatic atrophy (Imaging)<br>Liver/biliary tree: Intra- and/or extra-hepatic biliary duct stricture<br>Other: Disease-related or treatment-related diabetes mellitus	Pancreas: Pancreatic atrophy (Imaging), Long-term pancreatic enzyme replacement due to exocrine insufficiency<br>Liver/biliary tree: Intra- and/or extra-hepatic biliary duct stricture<br>Other: Disease-related or treatment-related diabetes mellitus	3	4	2	2	Disease Stable-Damage Increase
**case38**	male	77	3m	**1**	**3**	Pancreas: Long-term pancreatic enzyme replacement due to exocrine insufficiency<br>Liver/biliary tree: Intra- and/or extra-hepatic biliary duct stricture<br>Other: Partial or total resection of organs due to IgG4-RD	Pancreas: Long-term pancreatic enzyme replacement due to exocrine insufficiency<br>Liver/biliary tree: Intra- and/or extra-hepatic biliary duct stricture<br>Other: Partial or total resection of organs due to IgG4-RD	3	3	2	2	Disease Stable-Damage Stable
**case39**	male	59	1yr	**1**	**3.5**	Lacrimal/salivary gland	Lacrimal/salivary gland	1	1	2	2	Disease Stable-Damage Stable
**case40**	male	66	2yrs	**0.5**	**4**	Orbits	Orbits, nose/nasal sinus	1	2	3	3	Disease Stable-Damage Increase

In Disease duration before treatment, different time units were used for better comprehension. **Abbreviations:** m, month(s); yr(s), year(s).

*** Case 19: A case scenario sample for IgG4-RD DI Scoring**

The patient is a 47-year-old male. He first visited the outpatient clinic for the enlargement of the submaxillary gland and multiple lymph nodes, as well as cough and abdominal discomfort. Physical examination revealed enlarged bilateral submandibular glands, along with cervical, supraclavicular, axillary, and supraglottic lymph nodes. Laboratory tests showed normal complete blood count and blood chemistry tests. His serum IgG level was 40g/L, and serum IgG4 level was 34000mg/L. Computer tomography indicated diffuse enlargement of pancreas and ground glass opacity in both lungs, accompanied with enlarged mediastinal and retroperitoneal lymph nodes. Submandibular gland pathology results supported the diagnosis of IgG4-related disease. Therefore, the patient was diagnosed with IgG4-related disease. On diagnosis, the organ involvement included bilateral submandibular glands, pancreas, lymph node, and lungs.

He received prednisone 40 mg per day and rituximab 1000 mg on day 1 and day 14 for induction remission, and his symptoms were significantly improved. One month after treatment, he was diagnosed with diabetes, which was considered glucocorticoid-related, and his glucocorticoid was tapered within 3 months. The patients reported no discomfort after 6 months of treatment, and the serum IgG4 level decreased to 6800mg/L. However, image examinations still showed persistent pancreas enlargement during follow-up visit. At 3.5 years after treatment, the patient revisited for recurrence of submandibular gland enlargement and new-onset steatorrhea. He had been using insulin to control his blood glucose level. His serum IgG4 level increased to 24000mg/L. Computer tomography indicated enlarged submandibular glands and diffuse enlarged pancreas.

Please evaluate the damage score at 6-month and 3.5-year of treatment according to IgG4-RD DI.

IgG4-RD DI Scoring:

At 6 months, this patient had glucocorticoid-related diabetes with no other persistent organ damage, his IgG4-RD DI score was 1 (treatment-related diabetes).

At 3.5 years, this patient still had glucocorticoid-related diabetes, with persistent pancreas enlargement confirmed by imaging. His submandibular gland enlargement had completely recovered at 6 months and remained stable until relapse at 3.5 years, therefore the enlargement of submandibular glands was not considered organ damage. Thus, his IgG4-RD DI score was 2 (treatment-related diabetes and persistent pancreas enlargement confirmed by imaging).

Supplemental Table 2. The scores of all raters between the damage stable and damage increased group.

	Damage Stable					Damage Increased					
	Mean	SD	Median	25th Percentile	75th Percentile	Mean	SD	Median	25th Percentile	75th Percentile	P value Nonparametric
rater1	0.00	0.00	0	0	0	1.30	0.73	1	1	1	0.0000
rater2	0.20	0.52	0	0	0	1.65	1.18	1	1	2	0.0001
rater3	0.10	0.31	0	0	0	1.35	0.75	1	1	2	0.0000
rater4	0.20	0.52	0	0	0	1.60	1.19	2	1	2	0.0002
rater5	0.30	0.47	0	0	1	1.20	1.11	1	0	2	0.0051
rater6	0.10	0.31	0	0	0	1.20	0.95	1	0.5	2	0.0002
rater7	0.00	0.46	0	0	0	1.50	1.05	1	1	2	0.0000
rater8	0.15	0.59	0	0	0	0.90	1.07	1	0	1	0.0072
rater9	0.25	0.55	0	0	0	1.40	1.10	1	1	2	0.0005
rater10	0.15	0.75	0	0	0.5	1.50	1.05	1	1	2	0.0003

	Damage Stable					Damage Increased					
	Mean	SD	Median	25th Percentile	75th Percentile	Mean	SD	Median	25th Percentile	75th Percentile	P value Nonparametric
rater11	0.00	0.00	0	0	0	1.30	0.73	1	1	1	0.0000
rater12	0.25	0.44	0	0	0.5	1.45	0.94	1	1	2	0.0001
rater13	0.15	0.59	0	0	0.5	1.20	1.32	1	0	2	0.0080
rater14	0.10	0.31	0	0	0	1.30	1.38	1	0	2	0.0008
rater15	0.20	0.62	0	0	0	1.70	0.92	2	1	2	0.0001
rater16	0.25	0.44	0	0	0.5	1.35	0.81	1	1	2	0.0001
rater17	0.40	0.60	0	0	1	1.40	1.14	1	0.5	2	0.0043
rater18	0.10	0.31	0	0	0	1.30	1.13	1	0.5	2	0.0002
rater19	0.45	0.69	0	0	1	2.05	1.57	2	1	3·5	0.0012
rater20	0.00	0.00	0	0	0	0.60	0.99	0	0	1	0.0037
rater21	0.10	0.45	0	0	0	1.30	1.30	1	0	2	0.0022
rater22	0.00	0.00	0	0	0	1.30	0.73	1	1	1	0.0000

	Damage Stable					Damage Increased					
	Mean	SD	Median	25th Percentile	75th Percentile	Mean	SD	Median	25th Percentile	75th Percentile	P value Nonparametric
rater23	0.25	0.44	0	0	0.5	1.05	1.00	1	0	1	0.0046
rater24	-0.05	0.39	0	0	0	1.00	1.26	1	0	1.5	0.0016
rater25	0.15	0.37	0	0	0	1.30	1.03	1	1	2	0.0002
rater26	0.15	0.37	0	0	0	1.30	1.03	1	0.5	2	0.0003
rater27	0.25	0.72	0	0	1	0.65	1.14	1	0	1	0.2313
rater28	0.05	0.22	0	0	0	0.95	1.00	1	0	1	0.0004
rater29	0.10	0.31	0	0	0	0.95	1.05	1	0	1.5	0.0018
rater30	0.20	0.41	0	0	0	1.10	1.07	1	0	2	0.0055
rater31	0.25	0.79	0	0	0.5	1.55	1.28	1	1	2	0.0011
rater32	0.10	0.31	0	0	0	1.25	1.12	1	0.5	2	0.0002
rater33	0.47	0.51	0	0	1	1.35	1.09	1	1	2	0.0075
rater34	0.05	0.22	0	0	0	1.30	1.03	1	0.5	2	0.0001

	Damage Stable					Damage Increased					
	Mean	SD	Median	25th Percentile	75th Percentile	Mean	SD	Median	25th Percentile	75th Percentile	P value Nonparametric
rater35	0.45	0.60	0	0	1	1.20	0.95	1	1	2	0.0078
rater36	0.15	0.49	0	0	0	1.20	0.89	1	1	2	0.0002
rater37	0.16	0.50	0	0	0	1.25	0.97	1	1	2	0.0002
rater38	0.00	0.33	0	0	0	1.05	1.19	1	0	1.5	0.0009
rater39	-0.05	0.39	0	0	0	1.30	1.13	1	0	2	0.0001
rater40	-0.10	0.55	0	0	0	1.25	1.21	1	0.5	2	0.0003
rater41	0.05	0.22	0	0	0	1.15	0.93	1	1	1	0.0000
rater42	0.05	0.22	0	0	0	1.25	0.85	1	1	2	0.0000
rater43	0.15	0.37	0	0	0	1.55	1.10	1	1	2	0.0001
rater44	0.05	0.22	0	0	0	1.30	1.26	1	0.5	2	0.0001
rater45	0.10	0.31	0	0	0	1.10	1.21	1	0	2	0.0017
rater46	0.05	0.22	0	0	0	1.10	1.07	1	0	2	0.0004

	Damage Stable					Damage Increased					
	Mean	SD	Median	25th Percentile	75th Percentile	Mean	SD	Median	25th Percentile	75th Percentile	P value Nonparametric
rater47	0.10	0.31	0	0	0	1.10	0.85	1	1	1	0.0001
rater48	0.00	0.00	0	0	0	1.30	0.73	1	1	1	0.0000

Supplemental Table 3. The sensitivity and specificity of all raters between the damage stable and increased group.

	Sensitivity	Specificity	Accuracy	TP	FP	FN	TN
rater1	1.00	1.00	1.00	20	0	0	20
rater2	0.90	0.85	0.88	18	2	3	17
rater3	0.90	0.90	0.90	18	2	2	18
rater4	0.80	0.85	0.83	16	4	3	17
rater5	0.70	0.70	0.70	14	6	6	14
rater6	0.75	0.90	0.83	15	5	2	18
rater7	0.85	0.90	0.88	17	3	2	18
rater8	0.65	0.85	0.75	13	7	3	17
rater9	0.80	0.80	0.80	16	4	4	16
rater10	0.85	0.75	0.80	17	3	5	15
rater11	1.00	1.00	1.00	20	0	0	20
rater12	0.90	0.75	0.83	18	2	5	15
rater13	0.65	0.75	0.70	13	7	5	15
rater14	0.65	0.90	0.78	13	7	2	18
rater15	0.90	0.80	0.85	18	2	4	16
rater16	0.90	0.75	0.83	18	2	5	15
rater17	0.75	0.65	0.70	15	5	7	13
rater18	0.75	0.90	0.83	15	5	2	18
rater19	0.80	0.65	0.73	16	4	7	13
rater20	0.40	1.00	0.70	8	12	0	20
rater21	0.60	0.85	0.73	12	8	3	17
rater22	1.00	1.00	1.00	20	0	0	20
rater23	0.70	0.75	0.73	14	6	5	15

	Sensitivity	Specificity	Accuracy	TP	FP	FN	TN
rater24	0.60	0.95	0.78	12	8	1	19
rater25	0.80	0.85	0.83	16	4	3	17
rater26	0.75	0.85	0.80	15	5	3	17
rater27	0.55	0.70	0.63	11	9	6	14
rater28	0.65	0.95	0.80	13	7	1	19
rater29	0.60	0.90	0.75	12	8	2	18
rater30	0.60	0.80	0.70	12	8	4	16
rater31	0.80	0.75	0.78	16	4	5	15
rater32	0.75	0.90	0.83	15	5	2	18
rater33	0.80	0.53	0.67	16	4	9	10
rater34	0.75	0.95	0.85	15	5	1	19
rater35	0.80	0.60	0.70	16	4	8	12
rater36	0.80	0.90	0.85	16	4	2	18
rater37	0.80	0.89	0.85	16	4	2	17
rater38	0.60	0.95	0.77	12	8	1	18
rater39	0.70	0.95	0.83	14	6	1	19
rater40	0.75	0.90	0.83	15	5	2	18
rater41	0.85	0.95	0.90	17	3	1	19
rater42	0.85	0.95	0.90	17	3	1	19
rater43	0.85	0.85	0.85	17	3	3	17
rater44	0.75	0.95	0.85	15	5	1	19
rater45	0.60	0.90	0.75	12	8	2	18
rater46	0.65	0.95	0.80	13	7	1	19
rater47	0.80	0.90	0.85	16	4	2	18
rater48	1.00	1.00	1.00	20	0	0	20
