# Supplementary material for: The development and initial validation of IgG4-related disease damage index: a consensus report from Chinese IgG4-RD Consortium
Source: RMD Open. 2024 Mar 8;10(1):e003938. doi: 10.1136/rmdopen-2023-003938 (PMC10928742; doi:10.1136/rmdopen-2023-003938)
Supplement: Supplementary data [file rmdopen-2023-003938supp001.pdf]

Appendix 1:

Instruction Manual of CIC IgG4-Related Disease Damage Index (IgG4-RD DI)

Recording of Damage

The IgG4-RD DI by the Chinese IgG4-RD Consortium (CIC IgG4-RD DI) is a cumulative index consisting of damages in 14 domains, including 13 organ systems and other damages which were not listed in the previous 13 organ systems, including disease related damages, treatment-related adverse effects, and malignancies. "Damage" refers to irreversible organ damage that has lasted at least 6 months since IgG4-RD was diagnosed.

IgG4-RD Damage Index (IgG4-RD DI)

Date form completed: (dd/mm/yyyy)
__/______/_____

Case Number: ____________

Scoring Rules:

1) This form refers to damages due to IgG4-RD, treatment-related adverse effects, and malignancies, ascertained by clinical assessment, regardless of disease activity, that has been lasted for more than 6 months unless otherwise stated.
2) Every item of each domain should be assessed and scored individually.
3) Causes of organ damages other than IgG4-RD or related to treatment should not be scored.
4) IgG4-RD DI is a cumulative assessment of organ damage, the score can only remain stable or deteriorate.

Item	Score
**1. Nervous system**	
Persistent/residual meninges thickening (Imaging)	1
Cranial or peripheral nerve damage (not including optic nerve)	1
**2. Pituitary gland**	
Anterior pituitary dysfunction	1
Central diabetes insipidus	1
**3. Orbits**	
Visual impairment (if blindness, rate 2)	1(2)
Diplopia or exophthalmos	1
**4. Lacrimal / Salivary gland**	
Dry mouth with objective evidence of xerostomia	1
Dry eyes with objective evidence of xerophthalmia	1
**5. Mastoid / Auris Media**	
Persistent mastoiditis (Imaging)	1
Hearing loss	1
**6. Nose/nasal sinus**	
Persistent nasal sinus/turbinal lesions (Imaging) with relevant clinical symptoms	1
**7. Thyroid gland**	
Hypothyroidism (exclusion of other causes)	1
Persistent enlargement of thyroid gland compressing bronchia	1

Item	Score
**8. Lung**	
Persistent lung fibrosis/pleural thickening/masses/bronchial lesion/pleural thickening (Imaging)	1
Pulmonary hypertension	1
Impaired lung function (FVC<80% or FEV_1<80% or DLCO <70% predicted values)	1
**9. Cardiovascular system**	
Luminal stenosis, or formation of aneurysms (Imaging), or in need of arterial surgery	1
Constrictive pericarditis	1
**10. Retroperitoneum/mediastinum**	
Remaining retroperitoneal or mediastinal masses	1
Hydronephrosis/long-term D-J tube placement (more than six months)	1
**11. Pancreas**	
Pancreatic atrophy, persistent enlargement, or pseudocyst (Imaging)	1
Long-term pancreatic enzyme replacement due to exocrine insufficiency	1
**12. Liver / Biliary tree**	
Intra- and/or extra-hepatic biliary duct stricture (Imaging)	1
Persistent elevation of liver enzyme/bile duct enzyme/bilirubin over twice the normal upper limit	1
Compensated cirrhosis (Child-Pugh grade A) or regional portal hypertension	1
Decompensated cirrhosis (Child-Pugh grade B/C)	2
**13. Kidney**	
Persistent renal parenchyma/pelvis masses (Imaging)	1
24-hour urinary protein > 0.5g	1
eGFR 30-60ml/(min·1.73m^2) (calculated by CKD-EPI)	2
eGFR 15-30ml/(min·1.73m^2), or need renal replacement therapy	3
**14. Others**	
Irreversible damage to other organs not listed above (accumulative if >one site)	1
New-onset Malignancy (accumulative in case of more than one kind of malignancy)	1
Disease-related or treatment-related cerebrovascular/Cardio-vascular accident (if more than once, rate 2)	1(2)
Persistent drug-related myelosuppression (more than 6 months)	1
Glucocorticoids-related Ischemic osteonecrosis	1
Glucocorticoids-related Osteoporosis with fractures or vertebral collapse	1
Cataracts caused by glucocorticoids	1
Disease-related or treatment-related diabetes mellitus	1

Item	Score
Partial or total resection of organs due to IgG4-RD (accumulative if > one site), including orbitotomy, pulmonary wedge resection, Whipple procedure, nephrectomy, pituitary surgery, thyroidectomy, etc.	1

Total IgG4-RD Damage Index Score: _______

Appendix 2:

Instruction Manual of CIC IgG4-RD Damage Index Glossary

Damage: Irreversible organ damage **that has lasted at least 6 months** since IgG4-RD was diagnosed.
Imaging: Imaging examinations used as objective measures during evaluation of IgG4-RD patients, including doppler ultrasonography, computed tomography (CT), magnetic resonance imaging (MRI), positron emission tomography computed tomography (PET-CT), echocardiography, angiography, etc.

1. **Nervous system**
   1.1 Meninges thickening: Documented by appropriate imaging examinations.
   1.2 Cranial or peripheral nerve damage: Neuropathy leading to motor or sensory dysfunction, excluding the optic nerve.
2. **Pituitary gland**
   2.1 Anterior pituitary dysfunction: Clinical manifestations due to deficient secretion of one or more anterior pituitary hormones, confirmed by laboratory tests and appropriate imaging examinations.
   2.2 Central diabetes insipidus: Polyuria, nocturia, and polydipsia, preferably verified by blood level of anti-diuretic hormone and appropriate imaging examinations.
3. **Orbits**
   3.1 Visual impairment / blindness: Reduced acuity of sight or restricted vision in either eye, due to involvement of eye muscle and/or optic nerve and/or pituitary gland. If there is complete vision loss in either eye, rate 2·
   3.2 Diplopia or exophthalmos: Double vision due to eye muscle and/or corresponding motor nerve involvement, or chronic swelling of eye muscles and/or retro-ocular soft tissues.
4. **Lacrimal / Salivary glands**
   4.1 Dry mouth: Oral dryness for at least 6 months, or a frequent need to drink liquids to aid in swallowing dry food. The objective evidence of xerostomia is unstimulated whole saliva (UWS) ≤ 0.1 mL/minutes.
   4.2 months, or use of tear substitutes more than 3 times per day. The objective evidence of xerophthalmia is defined as ocular staining score (OSS) ≥5 and/or Schirmer's test (performed without anesthesia) ≤5 mm/5 minutes.
5. **Mastoid / Auris Media**
   5.1 Persistent mastoiditis: Chronic inflammation of middle ear with otorrhea, preferably confirmed appropriate imaging examinations.
   5.2 Hearing loss: Loss of hearing acuity due to middle ear involvement or auditory nerve/cochlear damage, preferably confirmed by audiometry.
6. **Nose/nasal sinus**
   6.1 Persistent nasal sinus/turbinal lesions: Documented by appropriate imaging examinations, with symptoms such as nasal blockage, hyposmia and nasal discharge.
7. **Thyroid gland**
   7.1 Hypothyroidism: Confirmed by laboratory testing of elevated serum thyroid-stimulating hormone (TSH) concentration and a decreased serum free thyroxine (T4) concentration, using locally determined methods.
   7.2 Persistent enlargement of the thyroid gland compressing bronchia: Physical signs of goiter, confirmed by appropriate imaging examinations.

8. Lung

8.1 Persistent lung fibrosis/masses /bronchial lesion/ pleural thickening: Documented by appropriate imaging examinations. A mass is defined as a solid lesion over 3.0 cm in diameter.

8.2 Pulmonary hypertension: Right ventricular prominence or loud P2, with echocardiography showed pulmonary artery systolic pressure ≥40 mmHg or mean pulmonary arterial pressure ≥25 mmHg at rest assessed by right heart catheterization.

8.3 Impaired lung function: Forced vital capacity (FVC) <80% or forced expiratory volume in one second (FEV_1) <80% or single breath diffusing capacity for carbon monoxide (DLCO) <70% predicted values).

9. Cardiovascular system

9.1 Artery luminal stenosis, or formation of aneurysms: Documented by appropriate imaging examinations. If a patient develops myocardial ischemia/infarction or typical angina, it is recommended to score item 14.3.

9.2 Constrictive pericarditis: Documented clinically and verified by appropriate imaging examinations.

10. **Retroperitoneum/mediastinum**

10.1 Remaining retroperitoneal or mediastinal masses: Documented by appropriate imaging examinations, with compression of surrounding organs. If the size of the lesion is significantly reduced after therapy, with minor remnants and without compressing the surrounding organs, score 0.

10.2 Hydronephrosis/long-term double-J-catheter drainage: Documented by appropriate imaging examinations. Long-term double-J-catheter drainage is defined as drainage last for > 6 months.

11. Pancreas

11.1 Pancreatic atrophy, persistent enlargement, or pseudocyst: Documented by appropriate imaging examinations.

11.2 Exocrine insufficiency: Malnutrition and/or malabsorption, requiring pancreatic enzyme replacement.

12. Liver / Biliary tree

12.1 Intra- and/or extrahepatic biliary duct stricture or persistent dilation: Documented by appropriate imaging examinations.

12.2 Persistent elevation of liver enzyme/bile duct enzyme/bilirubin over 2 times the normal upper limit: According to locally determined methods.

12.3 Liver cirrhosis: Confirmed by clinical manifestations, physical examinations, laboratory tests and appropriate imaging examinations, evaluated by Child-Pugh classification.

13. Kidney

13.1 Persistent renal parenchyma/pelvis masses: Documented by appropriate imaging examinations.

13.2 24-hour urinary protein > 0.5g: According to locally determined methods.

13.3 Estimated glomerular filtration rate (eGFR): Calculated by the 2021 chronic kidney disease epidemiology (CKD-EPI) creatinine equation.

13.4 Renal replacement therapy: Short-term dialysis less than 6 months, score 0.

14. Others

14.1 Damage to organs not listed above, e.g., prostate, mesentery, skin, etc.; accumulative if >1 site.

14.2 Malignancies: Newly onset and diagnosed after the diagnosis of IgG4-RD; the score is accumulative if there are multiple kinds of malignancies.

14.3 Disease- or treatment-related cerebrovascular/cardiovascular accident: Stroke (clinically or

documented by appropriate imaging examinations), leading to paresis, paralysis, and/or other disability. Angina, coronary artery bypass, or myocardial infarction (ever, confirmed by electrocardiograph and/or myocardial enzyme tests) related to IgG4-RD and/or treatment. Rate 2 if there are multiple accidents.

14.4 Myelosuppression: Leukopenia (white blood cells <4 $\times 10^9$/L) or thrombocytopenia (platelets <100 $\times 10^9$/L) or anemia (hemoglobin <100 g/L), confirmed by common blood counts; secondary to medication.

14.5 Ischemic osteonecrosis: Documented by appropriate imaging examinations; since the diagnosis of IgG4-RD.

14.6 Osteoporosis with fractures or vertebral collapse: By history confirmed on imaging.

14.7 Cataracts: Lens opacity confirmed by ophthalmoscopy secondary to glucocorticoids treatment.

14.8 Diabetes mellitus: Newly onset diabetes after diagnosis or treatment of IgG4-RD, requiring therapy.

14.9 Partial or total resection of organs due to IgG4-RD: The score is accumulative if there are multiple resection sites. If the same organ received resection for more than one time, score 1.
